# Establishing a consensus for the hallmarks of cancer based on gene ontology and pathway annotations

**DOI:** 10.1186/s12859-021-04105-8

**Published:** 2021-04-06

**Authors:** Yi Chen, Fons. J. Verbeek, Katherine Wolstencroft

**Affiliations:** The Leiden Institute of Advanced Computer Science (LIACS), Snellius Gebouw, Niels Bohrweg 1, Leiden, The Netherlands

**Keywords:** Gene ontolog, The hallmarks of cancer, Semantic similarity, Co-expression network

## Abstract

**Background:**

The hallmarks of cancer provide a highly cited and well-used conceptual framework for describing the processes involved in cancer cell development and tumourigenesis. However, methods for translating these high-level concepts into data-level associations between hallmarks and genes (for high throughput analysis), vary widely between studies. The examination of different strategies to associate and map cancer hallmarks reveals significant differences, but also consensus.

**Results:**

Here we present the results of a comparative analysis of cancer hallmark mapping strategies, based on Gene Ontology and biological pathway annotation, from different studies. By analysing the semantic similarity between annotations, and the resulting gene set overlap, we identify emerging consensus knowledge. In addition, we analyse the differences between hallmark and gene set associations using Weighted Gene Co-expression Network Analysis and enrichment analysis.

**Conclusions:**

Reaching a community-wide consensus on how to identify cancer hallmark activity from research data would enable more systematic data integration and comparison between studies. These results highlight the current state of the consensus and offer a starting point for further convergence. In addition, we show how a lack of consensus can lead to large differences in the biological interpretation of downstream analyses and discuss the challenges of annotating changing and accumulating biological data, using intermediate knowledge resources that are also changing over time.

**Supplementary Information:**

The online version contains supplementary material available at 10.1186/s12859-021-04105-8.

## Introduction

The hallmarks of cancer, presented initially in 2000 and updated in 2011 [1, 2], provides a conceptual framework for describing the process of tumorigenesis. The hallmarks suggest all cancer cells should have 10 essential molecular characteristics: (1) sustaining proliferative signaling, (2) evading growth suppressor, (3) resisting cell death, (4) enabling replicative immortality, (5) inducing angiogenesis, (6) activating invasion and metastasis, (7) genome instability and mutation, (8) tumor promoting inflammation, (9) deregulating cellular energetic and (10) avoiding immune destruction. Since the theory was proposed, it has been widely used for interpreting cancer research results, particularly in large-scale, big data studies where whole genome and transcriptome data are compared [3–5]. To date, the two Hallmarks of Cancer papers have been cited over 83,000 times [6], showing the utility of the hallmarks for describing cancer research results.

However, although the Hallmarks of Cancer are widely used to describe results, they describe cellular processes at a conceptual level, and the interpretation of these concepts, and also the methods for interpretation, vary between studies. In order to reach conclusions about the presence or absence of a hallmark process, researchers associate hallmarks with genes, or biological pathways or functional properties at the data level [5]. If these associations are made explicit, studies can be compared with one another and can be reproduced. If this information is omitted, comparisons are more difficult and studies are less reproducible [7]. The aim of this study is to identify previous attempts to explicitly associate cancer hallmarks with genes and functional annotation at the data level, and to assess the similarity of these interpretations. If we can identify and further develop consensus for data-level descriptions of cancer hallmarks, we will enable a more systematic use of the hallmark concepts and therefore enable a better understanding of the similarities and differences between cancer research results.

Previous attempts to formalise the descriptions of cancer hallmarks at the data level have followed a number of approaches. Text mining approaches have attempted to identify keyword matches to hallmarks from the literature [8], functional annotation approaches have attempted to use the Gene Ontology or biological pathway resources as intermediate knowledge sources to describe the hallmarks [5, 9, 10], and curation approaches have attempted to assess the involvement of individual genes that have previously been causally linked to cancer by expert analysis of the literature. The most extensive example of this final approach is the COSMIC database effort to manually describe hallmark characteristics for all genes in the cancer gene census [11]. Currently, 30% of the census has been described, so this represents ongoing work.

The most popular approach for defining cancer hallmarks is to use an intermediate knowledge resource. Using well-annotated intermediate knowledge resources, such as the Gene Ontology, or biological pathways, to classify and organise data is a well-established bioinformatics approach to data integration [12, 13]. However, it is not without challenges. There are a large number of biological pathway resources available. If different studies use different pathway resources, how comparable are the results? It has previously been shown that mapping between pathway resources is difficult and that selecting different pathway resources can significantly affect the results of enrichment analyses [14–16]. Consolidation between pathway resources, using pathway ontologies, such as [17], or by curating mappings between pathways [18] can mitigate these problems. If the Gene Ontology (GO) is used as an intermediate knowledge resource, the process of assigning Gene ontology terms to specific hallmarks can vary. In most studies, this activity is driven by domain experts, but the breadth of the cancer research domain can easily lead to bias in any individual study. Another problem with using GO is that it evolves quickly. The structure of GO changes and so do the numbers of genes annotated with any given term [19]. Associations between cancer hallmarks and GO terms may therefore not remain valid over time. As our understanding of biology in general, as well as cancer biology changes, associations between the cancer hallmarks and functional annotations must evolve to keep pace. In this study, we assess the differences and consensus between studies that have attempted to formalise the descriptions of the cancer hallmarks at the data level. From the literature, we have identified only 5 cases where the associations between hallmarks and GO terms, or hallmarks and biological pathways have been made explicit [5, 9, 10, 20, 21]. We refer to these associations as ‘mapping schemes’. Publications were only included if they contained a list of mappings to at least 7 of 10 cancer hallmarks, if the association procedure was described, and if the date of publication was after the 2011 hallmarks paper. From the five papers, four used the Gene Ontology and one used biological pathways for hallmark associations. One publication [9] used both GO and biological pathways, but the pathways were defined using a resource that is now obsolete, so the pathway definitions were omitted.

It is clear that other studies have also used similar associations, but without an explicit description of the mapping between hallmarks and functional annotation terms, they cannot reliably be reproduced for the comparison [22–26].

We compared the Gene Ontology and biological pathway terms selected to represent individual cancer hallmarks, both directly and by analysing their semantic similarity. In addition, we examined the differences between the sets of genes that were annotated with the selected GO terms and biological pathways, which we name ‘Hallmark Genes’.

In order to assess the impact of the differences between Hallmark Gene sets, (and further our understanding of the consensus), we compare downstream results by performing Weighted Gene Co-expression Network Analysis (WGCNA) [27] and enrichment analyses with prognostic cancer genes from the TCGA [28]. If the hallmarks of cancer represent the process of tumorigenesis, genes that show changes in expression that are prognostic for patient survival may have direct involvement in this process as ‘drivers’, or maybe closely associated ‘passengers’ [29]. Genes that are classified as both prognostic and hallmark genes would be expected to play more important roles in co-expression networks, so the ratios of genes classified as prognostic, hallmark and prognostic-hallmark were compared.

Finally, we investigated the structural changes to the Gene Ontology hierarchy in the time periods between publications to explore the role of GO evolution in differences between mapping schemes.

The results of these analyses provide a clearer picture of the consensus knowledge that exists for cancer hallmark annotation. By identifying this consensus, we create a common foundation for understanding the hallmark concepts at the data level. In doing so we highlight the challenges of integrating accumulated and distributed biological knowledge over time and also the importance of data provenance for the annotation of biological information for reproducible informatics results.

## Results

In this study we compared 5 different mapping schemes. 4 mapping schemes that use GO, named GO1 [9], GO2 [10], GO3 [20] and GO4 [21], and 1 pathway mapping scheme, named PW1 [5]. All provided explicit lists of GO and pathway identifiers used for hallmark annotation, and all described 7 or more hallmarks. For most methods presented, GO terms and pathways were assigned to hallmarks by focus groups of domain experts.

### Similarities between gene ontology terms

**Fig. 1:**
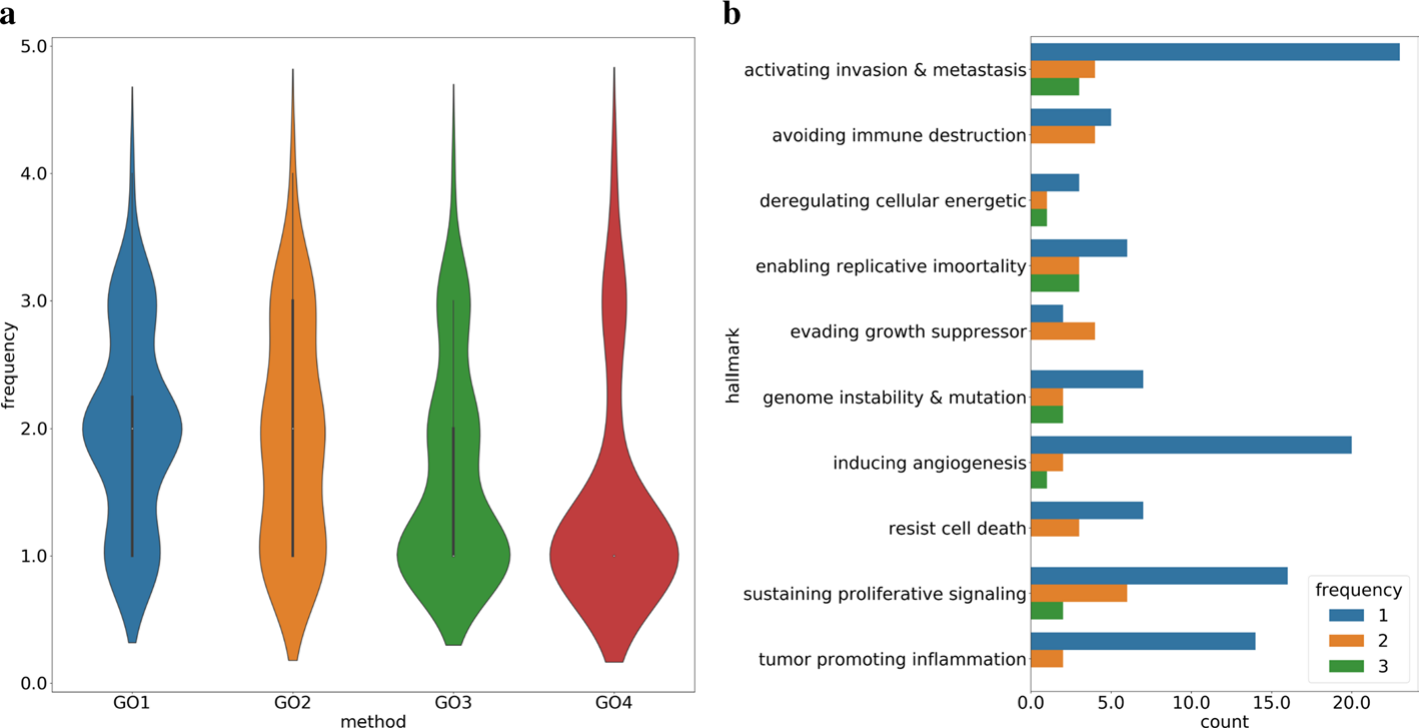
**a** Frequency of selection of GO terms from different schemes. **b** Frequency of selection of GO terms for individual cancer hallmarks. The x-axis represents the number of GO terms. The Y-axis represents individual cancer hallmarks. Bars coloured in different color represent how frequently it was selected by mapping methods to annotate this cancer hallmark

To investigate the consensus and divergence between different cancer hallmark mapping schemes, we first identified which Gene Ontology terms were consistently selected by different GO mapping schemes to annotate cancer hallmarks. As shown in Fig. 1a, most terms were not selected by all mapping schemes and many were unique to one scheme. In GO3 57.9% of terms were unique and in GO4 77.1% were unique. Only one term, ‘Negative Regulation of cell Cycle’ (GO:0045786), was selected by all 4 schemes. although it was mapped to different cancer hallmarks. In GO1 and GO2, it was mapped to ‘Evading Growth Suppressor’ while in GO3 and GO4, it was mapped to ‘Sustaining Proliferative Signaling’.

Figure 1b shows the number of GO terms shared between mapping schemes for each cancer hallmark. For most hallmarks, the majority of GO terms were only selected by 1 or 2 schemes, and a small proportion of terms were selected by 3 schemes. For ‘activating Invasion and metastasis’, more than 80% of terms were unique to one mapping scheme.

In contrast, although only twelve GO terms were selected by any scheme for the hallmark ‘enabling replicative signaling’, 3 out of 12 were selected by 3 schemes, showing a larger consensus than other hallmarks. For the hallmark ‘evading growth suppressor’, 4 of 6 terms were selected by GO1, and GO2, which indicates good consensus, but GO3 and GO4 did not provide any mapping terms for this hallmark, so the level of consensus is undetermined.

Despite the differences between mapping schemes and the inconsistencies concerning which GO terms should be used to annotate which hallmark, a degree of consensus was identified and indicates a core of shared knowledge.

### Analysing the divergence and consensus in hallmark gene sets

Of the five hallmark mapping schemes we identified for this study, four used the Gene Ontology as an intermediate knowledge resource, and only one used biological pathways from KEGG [30] and the MSigDB canonical pathways data set [31]. To directly compare these annotations, we either had to make use of existing mapping between GO and pathway resources, or we had to look at the intersection of the genes annotated with each resource. The former method is problematic due to the difference in the granularity of annotation (as reactions in pathways are typically mapped to GO, rather than individual gene product functions in pathways) . The latter is advantageous in this particular case because the authors of the pathway mapping method provided a full list of genes included in the biological pathways selected at the time of writing and could therefore be used for direct comparison.

**Fig. 2:**
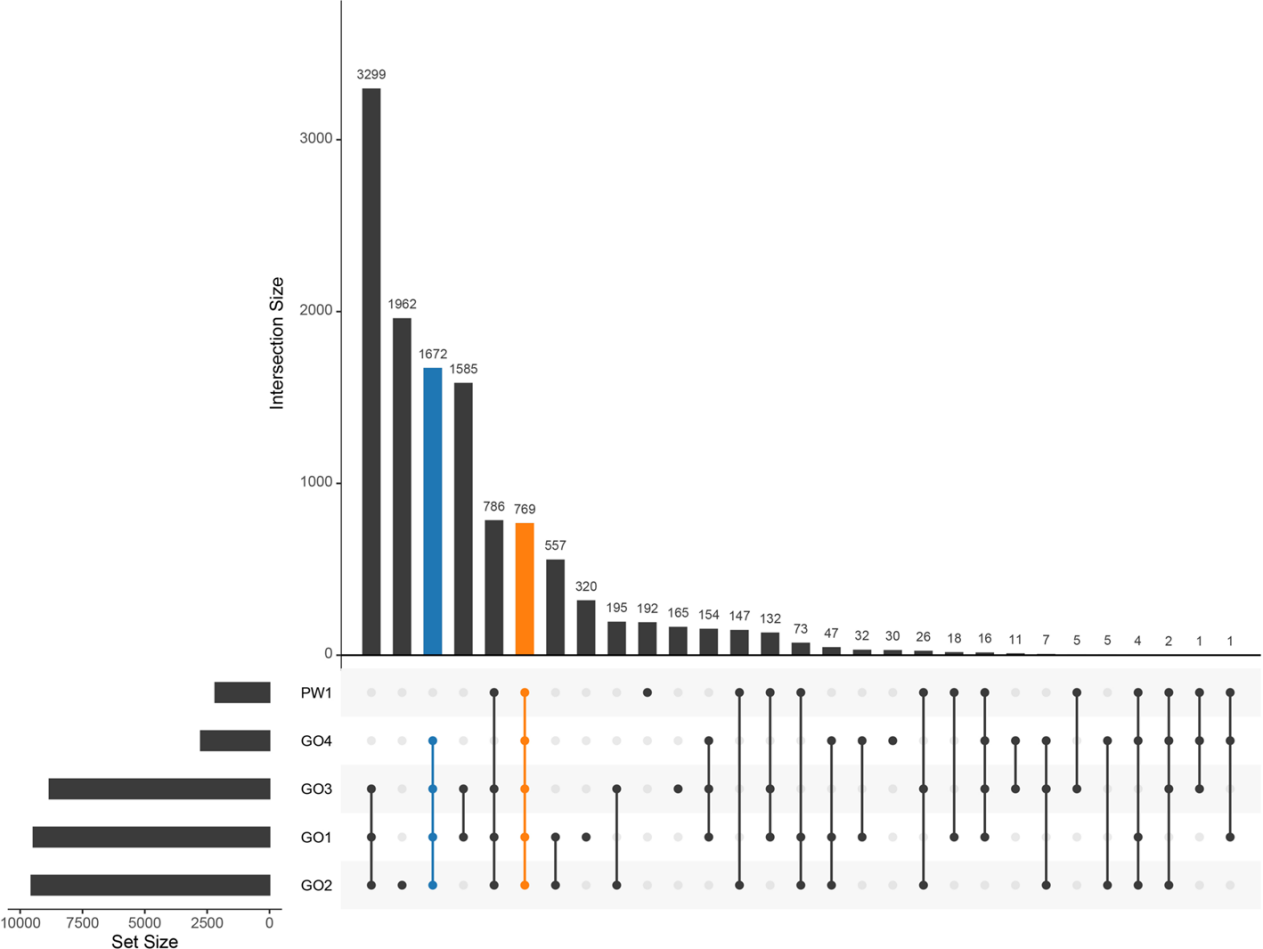
Hallmark Gene Set Comparison. The upset plot shows the number of genes in each hallmark gene set and their intersections. The orange and blue lines represent genes shared by GO mapping schemes and all mapping schemes respectively

The upset plot (Fig. 2) shows the intersections and differences between hallmark gene sets. Only 769 genes were identified as hallmark genes in all cases, and there was a marked difference in size between the gene sets. 2171 genes were identified as hallmark genes in PW1, while the smallest gene set generated by GO mapping schemes was 2752. The other three GO schemes generated sets of more than 8800 genes. This may indicate a greater specificity for PW1 and GO4, but as they were not found to be subsets of the larger hallmark gene sets, this does not fully explain the discrepancy.

The number of annotations for selected GO terms from GO4 were significantly lower than for the other schemes (Additional file 1) , demonstrating that more specific GO terms were used for hallmark definitions. We therefore suggest GO4 shows a greater specificity of term selection. In addition, however, GO terms corresponding to the hallmarks ‘Avoiding Immune Destruction’ and ‘Evading Growth Suppressor’ were not defined for GO4. The incomplete definition of the hallmarks could also contribute to the difference in data set size. The GO2 hallmark gene set has a similar size to GO1 and GO3, but it has more than 2000 unique genes. GO1 has 320 unique genes and GO3 has 165.

When hallmark gene sets were examined at the level of individual hallmarks, consensus and differences were observed (Additional file 2) . For ‘resisting cell death’, for example, GO1 and GO3 share the same gene set, but significant differences were observed in other hallmarks. For example, in ‘activating invasion and metastasis’, although GO2 has a larger hallmark gene set size, it has 1000 fewer genes for this hallmark than GO3. Based on these results, we conclude that significantly different hallmark gene sets are generated when different mapping schemes are utilized, which demonstrates the importance of reaching consensus knowledge for effective comparisons between studies that use the hallmark concepts to describe cancer research results.

It was not possible to include PW1 in this comparison as the authors of the study did not explicitly state which biological pathway related to which cancer hallmark. We could infer this for many of the pathways and we observed that multiple hallmarks may be represented by some pathways, but this is an interpretation of results, rather than a reuse of stated results and so was omitted.

### Impact of hallmark mapping strategies on downstream analyses

The results of comparing GO terms and hallmark genes have shown both consensus and difference. We therefore investigated the impact of using different mapping schemes on the results of downstream omics analyses.

#### Prognostic genes and hallmarks

**Fig. 3:**
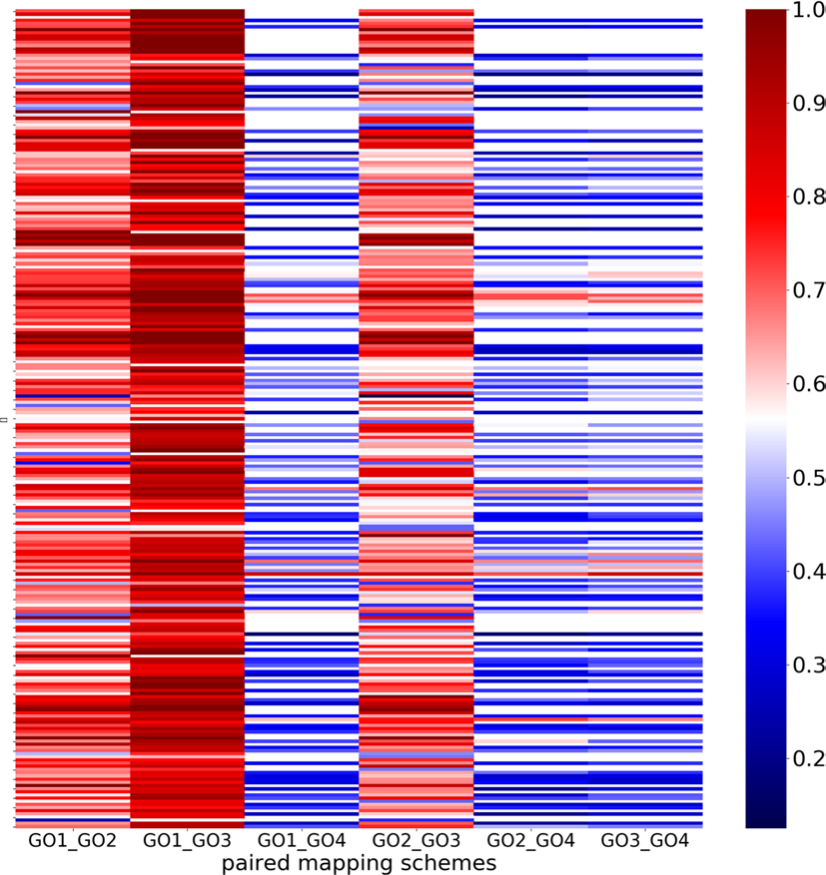
Pairwise comparison of groups of prognostic-hallmark genes identified in the same cancers between mapping schemes. Each block represents the overlap of a group of prognostic-hallmark genes shared by multiple cancer types with different mapping schemes. The X-axis shows pairwise comparison of mapping schemes. The color of each block represents the similarity scores of the same group of genes using the Jaccard index. Red represents a higher score and Blue represents a lower score

Survival analysis is often used to indicate the importance of genes for specific cancer types. By studying large numbers of individual cases, genes whose expression are prognostic for an unfavourable outcome can be identified. As hallmark genes are involved in the biological activities that promote cancer development, it is expected that prognostic genes would either be hallmark genes themselves or be co-expressed with hallmark genes [5]. As different mapping schemes would generate different hallmark gene sets, investigating the varying overlap in prognostic and hallmark genes would highlight the consequences of different hallmark definitions. Here we identified the overlap between prognostic and hallmark genes for 17 cancer types using different mapping schemes (referred to as prognostic-hallmark genes) . The prognostic gene data was taken from PW1 [5]. Prognostic-hallmark genes shared between multiple cancer types were identified. The impact of selecting different mapping schemes was assessed by pairwise comparisons where there were 5 or more shared genes. The Jaccard Index of prognostic-hallmark gene groups was calculated. Figure 3 shows the results.

Figure 3 shows the similarities between groups of prognostic-hallmark genes in different mapping schemes. As expected, none of the 6 comparisons showed complete consensus, reflecting the differences between hallmark gene sets. Comparisons between GO4 and the other three schemes were the most dissimilar, with the absence of any genes shared between some cancers (e.g. between Renal Cancer, Stomach Cancer and Endometrial Cancer) . GO1 and GO3 were grouped closest together, with the highest Jaccard index scores. Comparisons between GO1 and GO2, GO2 and GO3 produced a moderate scores. The full list of results can be found in Additional file 3.

#### Cancer hallmarks enrichment and co-expression networks

To further investigate the relationship between prognostic genes and cancer hallmark mapping schemes, we examined the co-expression networks of prognostic and hallmark genes and their enrichment in breast cancer. As hallmark gene sets are different, the prognostic-hallmark gene sets also differ in size and member genes. In this study, 294, 277, 289 and 91 prognostic-hallmark genes were identified for GO1 to GO4, respectively. By using the breast cancer transcriptome data from the TCGA Genomics Data Commons (GDC) data portal (https://portal.gdc.cancer.gov/), a gene co-expression network was constructed using the Weighted Gene Co-Expression Network Analysis (WGCNA) method, using the R package WGCNA [27].

**Fig. 4:**
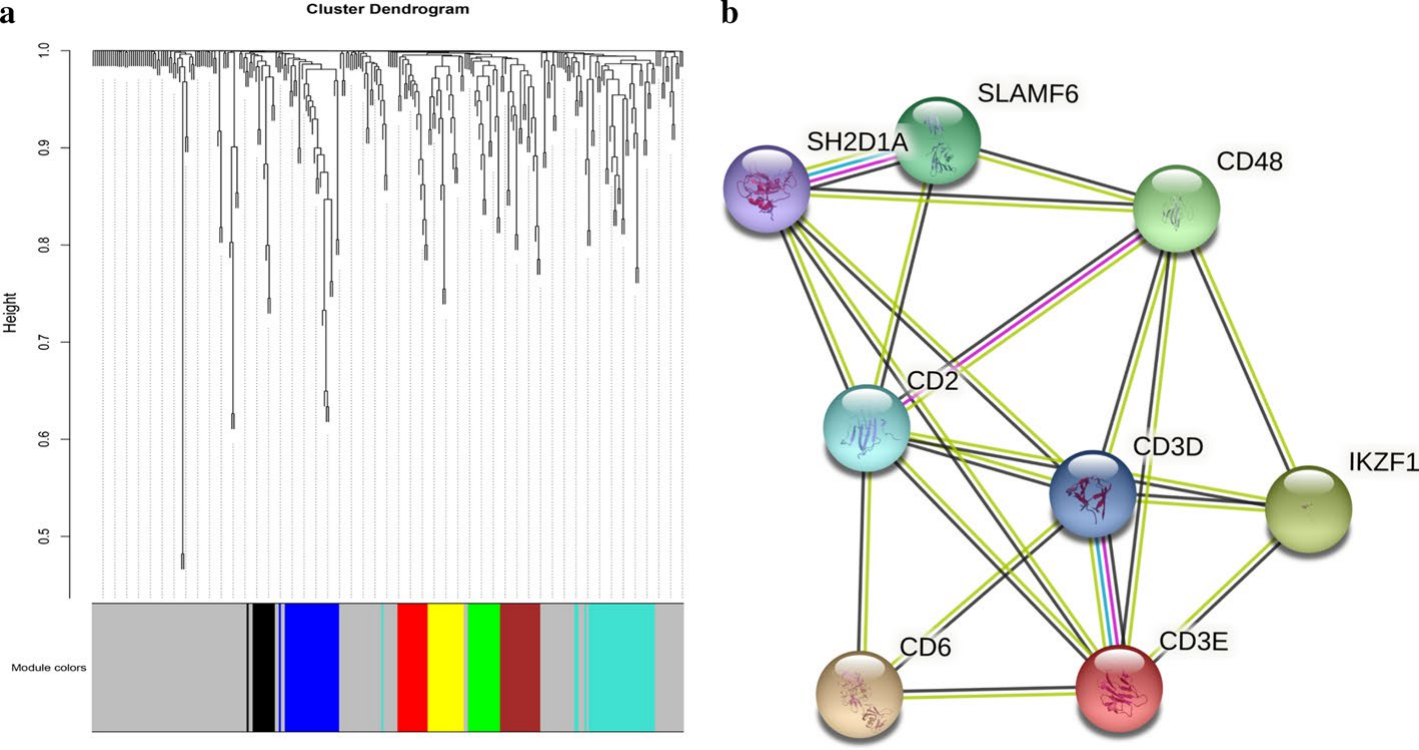
**a** Network analysis of prognostic hallmark gene expression in breast cancer with GO1 schemes identifies distinct modules of closely interconnected genes. **b** PPI network of hub genes of Module GO3_2, GO2_3 and GO1_2

In the WGCNA analysis, we identified 7 modules for GO1, 6 modules for GO2 and GO3 and 1 module for GO4 (Fig. 4a and Additional file 4: a–c). As only 91 genes were defined as prognostic-hallmark genes in GO4, while there were more than 200 genes in the other 3 schemes, it was reasonable that fewer modules were identified in GO4. For each module, hub genes were identified based on their intra-modular connectivity and an enrichment analysis was conducted to find associated biological activities with g:profiler. 5 genes with the highest intra-modular connectivity in each module were designated as hub genes. We identified a high overlap between modules from different mapping schemes. Module 7 in GO1 (GO1_7) and module 6 in GO3 (GO3_6) have the same hub gene sets. Similarly, GO3_2, GO2_3 and GO1_2 also had a highly overlapped hub gene set (Table 1). Further investigation of the proteins encoded by hub genes revealed that they were closely related, according to protein–protein interaction (PPI) data obtained from the String database [32] (Fig. 4b). For example, both gene CD3E (ENSG00000198851) and CD3D (ENSG00000167286) are part of the TCR-CD3 complex located on T-lymphocyte cell surface and are involved in adaptive immune response. CD3E is a hub gene in all 3 modules and the CD3D is a hub gene in module GO1_2 and GO3_2.

**Table 1:** Hub genes of modules

Gene id	Gene name	Function	modules
ENSG00000013725	CD6	Mediates cell-cell contacts and regulates T-cell responses via its interaction with ALCAM/CD166	GO3_2, GO1_2
ENSG00000116824	CD2	Mediate adhesion between T-cells and other cell types	GO3_2, GO2_3, GO1_2
ENSG00000167286	CD3D	Part of the TCR-CD3 complex,plays an essential role in adaptive immune response	GO3_2, GO1_2
ENSG00000198851	CD3E	Part of the TCR-CD3 complex,plays an essential role in adaptive immune response	GO3_2, GO1_2
ENSG00000117091	CD48	Facilitate interaction between activated lymphocytes and involved in regulating T-cell activation	GO3_2, GO2_3, GO1_2
ENSG00000185811	IKZF1	Plays a role in the development of lymphocytes, B- and T-cells	GO2_3
ENSG00000162739	SLAMF6	Associated with innate immune system and class I MHC mediated antigen processing and presentation	GO2_3
ENSG00000183918	SH2D1A	Plays a major role in the bidirectional stimulation of T and B cells	GO1_2

**Fig. 5:**
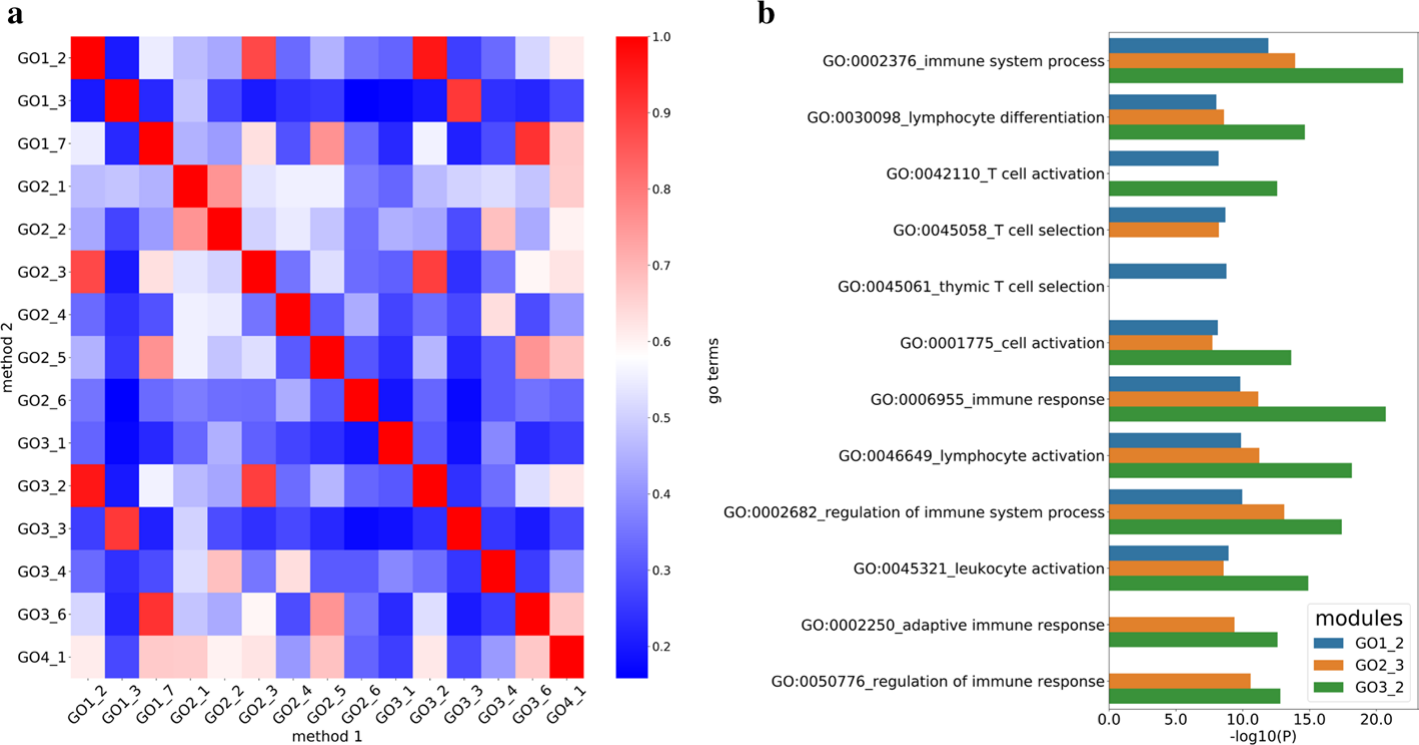
**a** Semantic similarity scores between modules. Red indicates high similarity and blue represents low similarity. **b** The top 10 significant GO terms (P< 0.05) in GO1_2, GO2_3 and GO3_2 modules

Enrichment analysis was performed on all modules and the functional similarity between them was evaluated. We selected all significant GO terms ($P<0.05$) and used the R package GOSemsim [33] for pairwise comparisons. As we can see in Fig. 5a, modules with highly overlapped hub genes have higher semantic similarity scores, corroborating the similarities in biological activity (e.g. GO3_2, GO2_3 and GO1_2). As shown Fig. 5b, all of the 3 modules were associated with the same GO terms related to the hallmark ‘avoiding immune destruction’, such as GO:0006955 ‘Immune response’ and GO:0002376 ‘immune system process’. Other terms such as GO:0045058 ‘T cell selection’ were enriched by all 3 modules, although it was not in the top 10 GO terms of GO3_2. Based on the enrichment results, we can conclude that the biological activities of the hallmark ‘avoiding immune destruction’ could be a leading factor in breast cancer. In contrast, for modules with low functional similarity, such as, module GO3_1 and GO2_5, different GO terms are enriched and therefore associated with different cancer hallmarks (Additional file 5). These results demonstrate that the initial mapping process of linking cancer hallmarks to GO terms has significant effects on downstream analyses and therefore overall conclusions. If the mapping process is not made explicit, comparisons between studies cannot be made. By reaching a consensus view on mapping, this situation can be improved.

### GO evolution in relation to difference between mapping schemes

The hallmarks mapping schemes under comparison were developed over the period of 7 years and therefore were developed using different versions of the Gene Ontology and associated annotation. Understanding which differences between mapping schemes were the result of topological or annotation changes to GO could therefore help to further refine consensus and make results and conclusions more comparable between studies.

Previous research has shown large changes in GO [19]. From 2004 to 2015, the number of terms in GO increased by 2.5 fold (from 16,139 to 40,810) and the number of GO terms used for annotations of human genes increased 3.8 fold (from 2972 to 11,403). Furthermore, the number of GO annotations for human genes changed from 19,615 to 109,152, increasing by 6.3 fold. The proportion of protein-coding human genes with at least 1 annotation changed from 32 to 65% and relationships between GO terms were also enriched and changed. 21,998 connections became 78,078 in the same period and 6833 relationships were removed. Other structural changes, such as, terms becoming obsolete or being merged into other terms also changed the hierarchical structure and information content of GO. According to the Archived data from the Gene Ontology Consortium (http://archive.geneontology.org/full/) , 1086 GO terms were made obsolete during this period. By constructing directed acyclic graphs (DAG) for each mapping scheme, based on selected GO terms and their neighbors, using Gene Ontology archived data at different time points, we evaluated the extent to which GO evolution contributed to the inconsistency between mapping schemes.

#### Structural differences in GO between mapping schemes

**Fig. 6:**
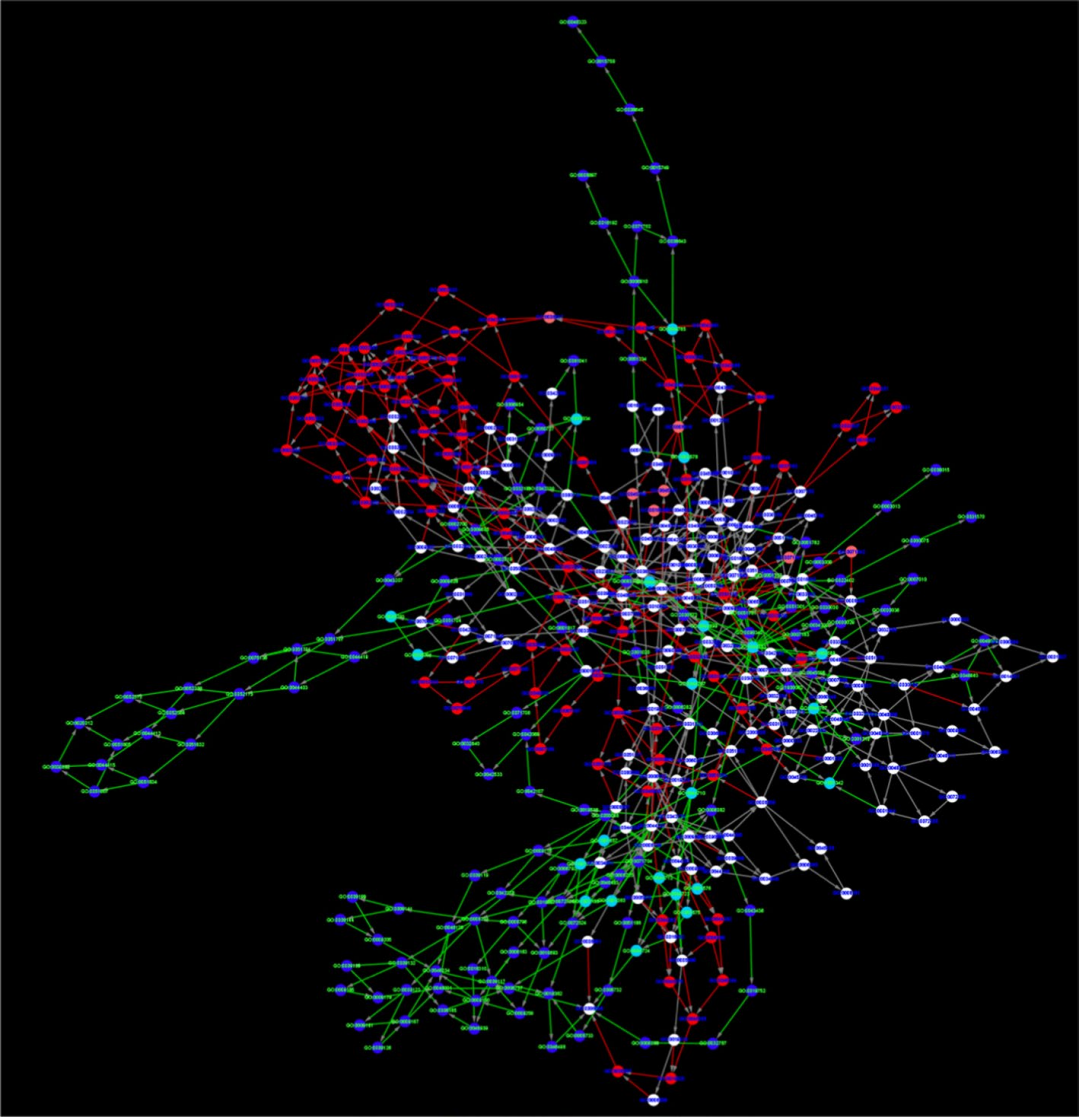
A comparison of the GO Biological Process topology of GO terms selected by GO1 (2016) and GO2 (2012), constructed from all selected GO terms and their first neighbours. Crimson nodes represent GO terms that existed in both time points but were only selected by GO2, while light red nodes represent GO terms which were obsoleted in 2016. Similarly, dark blue nodes represent GO terms that existed in both 2012 and 2016 but were only selected by GO1 and light blue nodes represent GO terms had not been created in 2012

GO2 is derived from a paper published in 2012, GO3 and GO4 were published in 2017, and GO1 was published in 2015. As none of the publications provided information on the version of GO used in their initial analyses, we selected evenly-spaced time points to study GO evolution across the whole period. To construct directed acyclic graphs (DAGs) to represent GO terms selected by a particular scheme, we first reconstructed the full GO hierarchical graph with the archived relationship data downloaded from Gene Ontology Consortium in Cytoscape [34]. For each mapping scheme, we generated a sub-graph of all GO terms selected and their neighbours, and made pairwise comparisons between each sub-graph. In Fig. 6, we compare the GO hierarchy graphs of GO1 (2016) and GO2 (2012). Crimson nodes represent GO terms that existed in both time points but were only selected by GO2, while light red nodes represent GO terms which were obsolete in 2016 and therefore unavailable for selection by GO1. Dark blue nodes represent GO terms that existed in both 2012 and 2016 but were only selected by GO1, and light blue nodes represent GO terms had not been created in 2012 and not available for selection by GO2, but were selected in 2016 by GO1. Figure 6 shows large differences between the DAGs for each scheme. Seven GO terms selected by GO2 were obsolete by 2016, and 20 terms selected by GO1 were not created in 2012, so structural differences played a role, but did not account for all differences. Similar observations were made in the comparison between GO3 and GO4 (Additional file 6). These results show that although there were structural changes to GO over the time period, the majority of terms were available for selection for each mapping scheme. This suggests structural differences were not the main factor and that differences in interpretation of the relationships between GO and the cancer hallmarks played a larger role. It is worth noting that the mapping scheme from GO2 is being used and maintained for further research by the authors of the GO2 paper and others. Despite all the changes to GO, there have been no updates to this mapping scheme since the initial publication.

An additional investigation into the changes to the numbers of annotation to GO terms over the same time period showed a general increase in the number of all annotations (Additional file 7). Changes in GO annotations increase the number of genes in a hallmark gene set. Mapping schemes that favour less specific GO terms for hallmark definitions therefore show a larger increase in hallmark gene set size. This is reflected in the results of downstream analyses, where we observe larger impacts on results.

### Consensus between methods

**Fig. 7:**
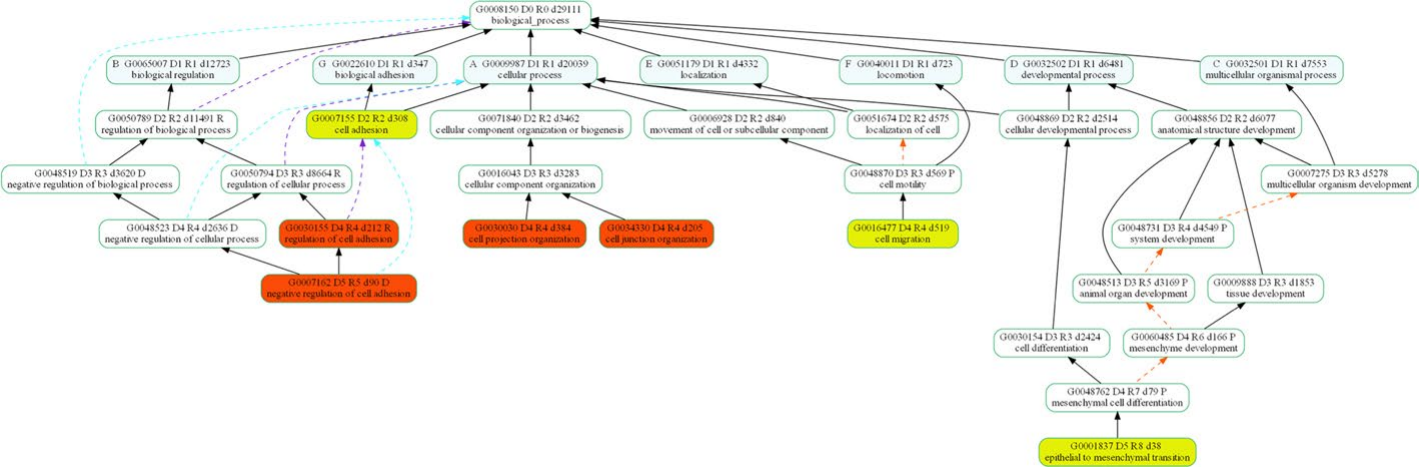
Visualization of the consensus GO terms for defining the cancer hallmark ‘Activating Invasion and Metastasis’. Nodes colored in red were selected by 3 schemes and nodes coloured in yellow represent GO terms selected by 4 methods

While the results of these analyses show inconsistencies between different mapping schemes, they also show consensus. From this consensus, we can identify a common understanding of functional cancer hallmark mapping, which could contribute to the creation of a systematic mapping method. The goal is to combine current consensus knowledge and maintain an active integration with GO and pathway resources as they change in the future. To investigate the consensus from all mapping methods, we combined GO and pathway results by identifying corresponding GO terms for the pathways selected by PW1. For pathways from the MSigDB canonical pathways data set, GO and pathway mapping has been specified and published by MSigDB and was used directly (Additional file 8). For pathways from KEGG, corresponding GO terms were not provided by KEGG directly. Therefore, corresponding GO terms were retrieved by identifying GO terms whose definitions were the most similar to the description of KEGG pathways. 14 GO terms were identified to represent the KEGG pathways. After correspondence between GO and pathways had been established, we used GO terms to describe and define the level of consensus across all five mapping schemes. For each cancer hallmark, GO terms selected by three or more mapping schemes were seen as consensus terms. In total, we identified 42 consensus GO terms across all hallmarks and we identified some degree of consensus for each hallmark (Additional file 9). Figure 7 shows the visualization of the hallmark ‘Activating Invasion and Metastasis’ where there were seven consensus terms. Nodes coloured in orange and yellow represent those selected by 3 and 4 methods respectively. Similarly, for the hallmark ‘Sustaining Proliferative Signaling’, there were also seven terms considered as consensus terms. These hallmarks show the most consensus. Other hallmarks showed much lower levels of agreement. For example, for ‘Inducing Angiogenesis’, only two terms were consensus terms, while 23 different terms were chosen across all mapping schemes to annotate this hallmark. A similar situation can be seen in ‘Tumor Promoting Inflammation’ where only two terms were consensus terms out of 16 terms selected across all methods. These results indicate that a refinement is required in the hallmark definitions, in order to establish a better consensus.

## Discussion

This study compares the differences between cancer hallmark functional annotation descriptions, developed for different purposes at different time-points. Although the hallmarks are in widespread use, the numbers of publications that explicitly describe the association between the hallmark concepts and biological molecules and/or functional annotations are limited. By building on the knowledge from these explicit associations, we explore the extent of existing consensus.

The core hallmark gene set identified at the intersection of compared mapping schemes is small, but it offers important information about where there is shared understanding. Genes in the core gene set are not all annotated with a small number of hallmarks, but spread across all ten hallmarks. This means that there is a partial shared understanding across all hallmarks and all mapping schemes. Similarly, when examining the consensus GO terms, there are consensus terms for every hallmark, although the amount of consensus varies. For the hallmark ‘Activating Invasion and Metastasis’, seven GO terms are consensus terms and three of them are selected by four mapping schemes. In contrast, the hallmark ‘Inducing Angiogenesis’, only has two consensus terms out of 23 terms selected across all mapping schemes. This suggests that the definition for this hallmark is currently insufficient and should be further refined.

The WGCNA and enrichment analysis results showed how the same transcriptomics data set could be interpreted in different ways, depending upon the initial selection of hallmark annotation definitions. Modules from different mapping schemes indicated enrichment by completely different hallmarks (Additional file 5 shows two modules unique to one mapping scheme), leading to different conclusions about which hallmarks were playing a role in breast cancer prognosis. These results highlight the importance of building consensus and enabling a more systematic annotation of hallmark activity.

Some modules showed high functional similarity between different mapping schemes, and also shared similar sets of hub genes. These collective results show a clear signature for the hallmark ‘Avoiding Immune Destruction’ across multiple mapping schemes, indicating the importance of this hallmark for prognosis in breast cancer. It is worth noting that we would not expect all hallmarks to be represented in the WGCNA results for only one cancer, but the fact that we have clear signatures for some hallmarks shows an association between the prognostic genes and the functional annotation that represents the hallmarks.

The consensus GO terms identified for each cancer hallmark show where there is a shared understanding of the hallmarks of cancer. They could therefore be the foundation for a more systematic approach to mapping cancer hallmarks to data via intermediate knowledge resources. In the longer term, the consensus could be the starting point for a broader community discussion and combined with other efforts. For example, the COSMIC data resource (Catalogue of Somatic Mutations in Cancer) [28] has undertaken a manual annotation approach to hallmark identification. For each gene in the COSMIC Gene Census, curators are manually extracting evidence for cancer hallmark involvement from the literature. To date, approximately 300 genes have been annotated out of 700. The process is slow and will take a number of years to complete, but COSMIC expects to identify hallmark activity for almost all genes in the census. When we compare the consensus GO terms from this analysis with those enriched in the Gene Census, the majority (31/42) are present and enriched, indicating that the consensus GO terms we have identified represent important consensus knowledge.

### Study limitations and future work

The fact that we only identified one example of a pathway-based approach that fitted our inclusion criteria, against four GO approaches, is a limitation for establishing pathway consensus. Nevertheless, both methods are being used and should therefore be considered here. The GO-generated hallmark gene sets were much larger than those of the pathway example and were caused by the inclusion of high-level terms from the GO hierarchy. Terms from higher levels have a larger number of genes annotated with them, but they also have a low information content and lack specificity. For example, terms such as, Immune Response (GO:0006955) or Cell Cycle (GO:0007049), were selected to define cancer hallmarks. Substituting these general terms with more specific descendant terms could make the GO term set more informative and reduce the overall number of gene products. However, the differences in term positions in the GO hierarchy cannot explain the differences between mapping schemes. Mapping schemes with smaller hallmark gene sets were not defined by descendants of the more general terms selected by mapping schemes with larger hallmark gene sets. This is highlighted by the low pairwise semantic similarity scores between mapping schemes.

A major limitation for analysing the semantic similarity between mapping schemes, however, is that current semantic similarity methods assume the same underlying knowledge structure. In reality, we were comparing annotations derived from different versions of the knowledge structure. For a fair comparison, we need to know if the same GO terms were available for each different study to select, or if some were only introduced later, or became obsolete between time-points. Our analysis of the GO topological structure revealed that most terms were available through the whole time-period, but that the researchers simply did not select the same (or similar) terms.

Rearrangements in higher level terms from the GO biological process hierarchy meant that relationships between many terms selected to represent hallmarks were altered significantly between time points. For example, in 2012 the term ‘death’ (GO:0016265) was a descendant of ‘Biological process’, and had one descendent, ‘cell death’ (GO:0008219). In March 2016, this term was made obsolete. The descendant term ‘Cell death’ was originally connected to the term ‘cellular process’ (GO:0009987) in the 2012 network, but by 2016, it had been removed and substituted with a linkage to the term ‘single-organism cellular process (GO:0044763)’, which was created after 2012. Alterations at high levels in GO affect the whole ontology structure and therefore have a large impact when calculating semantic distance or information content between sets of GO terms. Current semantic similarity measures do not take such changes into account and we propose the development of new approaches to semantic similarity that will include changes to the ontology structure over time.

Building a shared understanding of the cancer hallmarks at the data level, enables more insight into comparisons between research results, particularly where high throughput studies are concerned. As we generate more data and invest further in personalised approaches to cancer therapy, it is more important to focus on systematic methods to organise, classify and compare results. Functional annotation is an essential component in most bioinformatics analyses. Collecting better provenance about the annotation process and being more explicit about underlying assumptions and associations can help us make the best use of those annotations in the changing landscape of biological knowledge.

## Methods

### Background to Publications Selected for Comparison

This study is based on the analysis of cancer hallmark mapping schemes from publications that explicitly describe the mapping between intermediate knowledge bases and the hallmarks. The motivations for hallmark mapping were different between these publications, but all attempted to identify a general hallmark representation. There were two common ways to link the hallmarks of cancer to biological molecules and data; (1) mapping to Gene Ontology terms and (2) mapping to biological pathways. Both types of annotation allow a direct link to individual genes. We considered publications dating from the second cancer hallmarks review paper [2], in order to take all 10 hallmarks into account, but we included publications that described 8 or more hallmarks. Most importantly, we only considered publications that provided lists of the terms used for mapping and also at least a brief description of the process of associating the terms to hallmarks. In total, four publications and one peer-reviewed poster satisfied these criteria. Ulhen et al [5] mapped hallmarks to specific biological pathways, referred to by KEGG [30] and MSigDB [31] identifiers and described in terms of the gene products found in the pathways. We refer to this mapping scheme as PW1. The other four mapping schemes were based on the association between gene ontology (GO) terms and cancer hallmarks, and are referred to as GO1 [9], GO2 [10], GO3 [20], GO4 [21], respectively. The mapping scheme created by Plaisier (GO2) [10] was also used and cited in 2016 [35] and Thorsson [36]. Although the GO mapping scheme is maintained by the authors at https://github.com/baliga-lab/sygnal/blob/master/R/goSimHallmarksOfCancer.R, it did not appear to change between these time-points. The full study workflow can be found in Additional file 10.

The hallmarks of cancer mapping schemes from each publication were developed using different methodologies and in accordance with the authors’ background knowledge and aims. Table 2 summarises the main differences.

**Table 2:** Summary of 5 mapping methods

Methods	Source	Experts involved	Size (pathway/GO)	Gene number	Hallmark included
GO1	Gene ontology	Yes	57 (2 obsolete)	9465	10 hallmarks
GO2	Gene ontology	Unknown	40 (2 obsolete)	9551	10 hallmarks
GO3	Gene ontology	Yes	67	8825	8 hallmarks
GO4	Gene ontology	Unknown	35	2751	8 hallmarks
PW1	MSigDB, KEGG	Yes	14	2171	10 hallmarks

For PW1, the gene products annotated with each selected biological pathway were recorded by the authors at the time of publication and were therefore used as they were at publication. they were also updated to their current representations, in order to observe the differences. For GO1–GO4, genes annotated with the selected terms (or their descendants) were not included in the publication data and were therefore reconstructed from archival GO data and current information. The genes currently annotated by selected Gene Ontology terms were identified using the human ENSEMBL database (version 99) [37] via biomart [38] and descendant terms were identified using QUICKGO [39]. The list of GO terms selected by each method and identified for each individual hallmark are available in Additional file 11. The full list of genes annotated by selected Gene Ontology terms and biological pathways are available in Additional file 12.

### Hallmark gene comparison

To examine the overlap between Hallmark Genes from different mapping schemes, an upset plot was constructed using the R package UpsetR [40]. In addition, the number of genes annotated to individual cancer hallmarks with different GO mapping schemes was also investigated. The bar chart was created using the Python package Seaborn [41].

### Prognostic genes

The definition and data for prognostic genes was taken from the PW1 publication [5] as they provided a list of prognostic genes for 17 different types of cancer types in detail (Additional file 13).

### Prognostic-hallmark genes in multiple cancers

Genes labelled as both prognostic genes and hallmark genes are named prognostic-hallmark genes. For each method, we classified genes into different groups based on the number of cancer types where they were exclusively labelled as prognostic-hallmark genes. Then for each group, we further classified them into subgroups based on cancer types where they were labelled as prognostic-hallmark genes. Subgroups with less than 5 genes were excluded. For each subgroup, if it existed in at least one method, it was included. For existing subgroups, we calculated the Jaccard Index to determine how different the subgroups were in pairwise comparisons of the mapping schemes. If a subgroup did not exist in 1 of 2 compared mapping schemes, the score was 0. Results were visualized by heatmap using the Python Package Seaborn [41].

### Gene co-expression and clustering

Breast cancer was selected for co-expression network analysis because there are more than 1000 breast cancer cases in the TCGA database (the 3rd largest), and it only has 582 prognostic genes. When constructing a co-expression network, a large number of cases can minimize correlation coefficient bias and a relatively small number of prognostic genes can help to minimise the number of prognostic genes that need to be excluded from the study due to an insufficient coefficient value. The RNA-Seq data was obtained from TCGA with HTSeq-FPKM values [28]. 1222 breast cancer samples were downloaded. Weighted Gene Co-expression Network Analysis (WGCNA) was utilized to construct prognostic hallmark gene co-expression networks [27]. During data processing, we log transformed the FPKM value, and then removed outliers by clustering samples. Next, after measuring the co-expression similarity using pearson correlation, we further transformed it into the weighted co-expression network by raising the co-expression similarity to a power picked by function ‘pickSoftThreshold’. As modules are defined as highly interconnected genes, we adopted automatic block-wise module detection function to cluster closely connected genes into modules.

### Hub gene detection and functional enrichment analysis

The hub genes in WGCNA modules are genes with the high intramodular connectivity. Modules of constructed networks are exported to Cytoscape, and hub genes of each modules are identified based on intramodular degree. 5 genes with highest degree are chosen. For module GO1_2 and Module GO3_6, 6 genes are selected as there are two genes meet the lowest degree requirements of hub genes in both modules. Functional enrichment analyses of modules were conducted using g:profiler [42]. GO terms with P value less than 0.05 were considered significant results. Only terms from the Biological Process hierarchy were considered.

### Functional similarity and protein–protein interaction network

The functional similarities between the modules were calculated in order to determine if there were close or distant relationships between enriched terms of different modules. The R package GOSemSim [33] was used with a best match average strategy (BMA). The annotation data used was the Genome wide annotation for the Human from Bioconductor [43]. The Wang method [44] was used to assess semantic similarity. Modules enriched in only 1 or 2 GO terms were not included. Protein–protein interaction networks were constructed using STRING [32]. Hub genes of modules with high functional similarities were used to construct the PPI network.

### GO evolution

To investigate the impact of changes to the structure of GO, we used the Gene Ontology Biological Process hierarchy and associated annotations at three time points (June 2012, June 2016, Jan 2021). Data including GO term relationships and gene product count was downloaded from the Gene Ontology Consortium (http://archive.geneontology.org/) [45].

### Comparison between different Versions of GO

We constructed two GO hierarchy graphs in Cytoscape [34] based on archived relationship data downloaded from the Gene Ontology Consortium corresponding to different time points. For GO1, GO3 and GO4 mapping schemes, we created 3 sub-graphs of selected GO terms from each mapping scheme, their first neighbors and the edges between them based on the 2016 graph. For GO2, we created a sub-graph in similar way based on the 2012 graph. The comparisons between sub-graphs was performed using DyNet [46]. White nodes and edges represent GO terms and relationships which are included in both networks while nodes and edges with color represent GO terms or relationships which are included in 1 of 2 networks. Dot plots shows the number of gene products annotated to different GO terms. They were created by using the Python package Seaborn [41].

### Consensus between methods

For individual cancer hallmarks, a consensus GO term is one that was selected by more than 3 methods. Visualization of consensus terms belonging to hallmarks was performed using GOA-tools [47]. GO terms selected by 3 and 4 schemes were colored orange and yellow respectively. Corresponding GO terms for MSigDB pathways are identified and mapped by the MSigDB database and are therefore used directly in our comparison. The KEGG pathway database does not provide an equivalent mapping to corresponding GO terms, so we derived these mappings by examining the most similar GO term definitions and KEGG pathway definitions.

### Data provenance

This study aims to compare multiple mapping schemes and data from multiple time points. In order to make the work here transparent and reproducible, the provenance of all data and tools are listed. The descendants of selected GO terms were identified using QuickGO API and downloaded in Jan 2021. Genes annotated to selected GO terms and their descendants were identified using Biomart with the Ensembl 102 [37] dataset. The version of GOsemsim [33] used for semantic similarity was 2.14.0 and the annotation dataset in GOsemsim was Homo Sapien from OrgDb, version 3.12 [43]. The underlying GO version for each of these tools was declared to be the latest version at the time of analysis, although the exact version number was not provided by tool documentation. The classification of prognostic genes for 17 cancer types was taken directly from Ulhen et al, 2017 [5]. RNA-Seq data for breast cancer co-expression network construction was downloaded from TCGA, v23.0, and published on the 7th April, 2020. Gene Ontology data for 2012 and 2016 was taken from the Gene Ontology archive, published in June 2012 and June 2016 respectively. Pathway data from PW1 was from MSigDB version 5.2.

## Supplementary information

**Additional file 1**. The annotation counts of GO terms from different mapping schemes. The boxplot presents the number of annotations belonging to selected GO terms. GO1, GO2, GO3 and GO4 represent 4 GO mapping schemes.

**Additional file 2**. The genes belong to individual cancer hallmarks with different mapping schemes and their intersections. The dots represent the number of genes attributed to individual cancer hallmarks and the bars represent intersections of all mapping schemes.

**Additional file 3**. Jaccard Index of prognostic-hallmark gene groups. Jaccard index score of the set of prognostic-hallmark genes annotated to the same cancer groups when applying paired mapping schemes.

**Additional file 4a–c**. The cluster dendrograms of prognostic hallmark gene sets from GO2, GO3 and GO4. Prognostic hallmark genes are clustered into different modules based on their co-expression similarity.

**Additional file 5**. The top 10 enriched GO terms of module GO3_1 and GO2_5. The bar plot shows the top 10 GO terms that module GO3_1 and GO2_5 enriched in, respectively.

**Additional file 6**. Network comparison between GO3 and GO4. The two networks were constructed by utilizing GO archived term-to-term relationship data. Network comparison was accomplished by using Dynet. Green nodes are GO terms only selected by GO3 while red nodes are GO terms only selected by GO4.

**Additional file 7**. The annotation counts of GO terms at different GO version. It presents annotation counts of all selected GO terms at 3 time points (2012,2016,2021).

**Additional file 8**. Correspondence between MSigDB pathways and GO terms. GO terms corresponding to MSigDB pathways were extracted directly from MSigDB.

**Additional file 9**. Consensus among all mapping schemes. GO terms selected by more than 2 schemes (without PW1)/3 (with PW1) are considered as consensus terms.

**Additional file 10**. Study workflow. It can be divided into 2 parts. The first part presents the comparison between different mapping methods and the investigation of the impact of using different mapping schemes on downstream analysis. The second part shows the process of investigating the impact of GO evolutions on the differences between mapping schemes.

**Additional file 11**. Full list of GO terms collected from papers. GO1, GO2, GO3 and GO4 represent 4 GO mapping schemes collected from 3 papers and 1 poster. Every page shows an individual cancer hallmark and GO terms which were assigned to it by different methods.

**Additional file 12**. List of hallmark genes. Hallmark genes of GO mapping methods were generated by finding genes annotated to GO term selected by each method. The GO data and gene annotation was downloaded in Jan 2021. The full list of genes annotated to pathways was provided in the literature’s supplementary files.

**Additional file 13**. Prognostic genes of 17 cancer types.

## Data Availability

All data generated or analysed during this study are included in this published article and its supplementary information files. In addition, data can be found in the FAIRDOMHub repository (https://fairdomhub.org/projects/223#investigations), and code is available in https://github.com/chestnzu1/BMC
