# Supplementary figures and images for: Establishing a consensus for the hallmarks of cancer based on gene ontology and pathway annotations
Source: BMC Bioinformatics. 2021 Apr 6;22:178. doi: 10.1186/s12859-021-04105-8 (PMC8025515; doi:10.1186/s12859-021-04105-8)

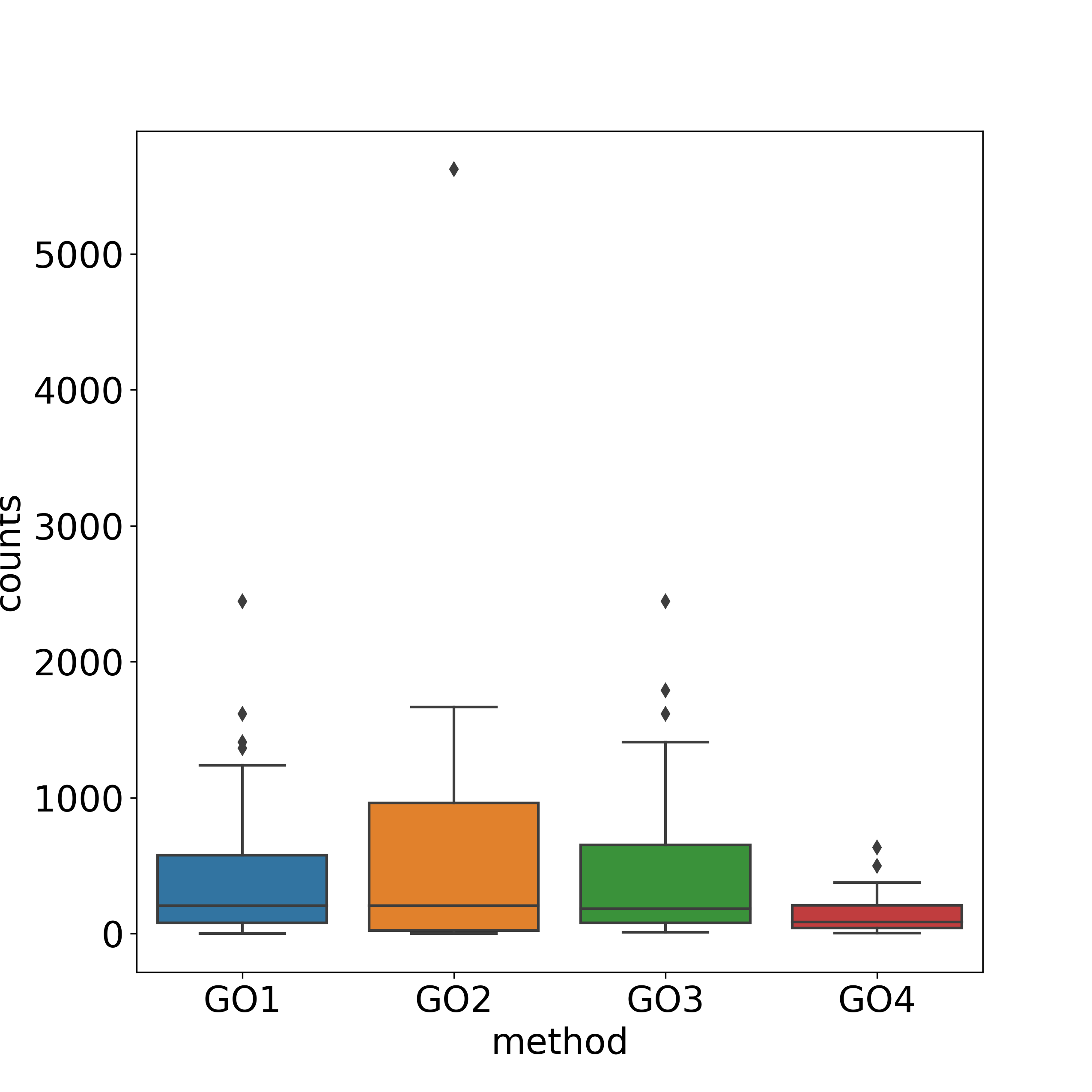

Supplement: Supplementary file 1 — Additional file 1. The annotation counts of GO terms from different mapping schemes. The boxplot presents the number of annotations belonging to selected GO terms. GO1, GO2, GO3 and GO4 represent 4 GO mapping schemes. [file 12859_2021_4105_MOESM1_ESM.png]

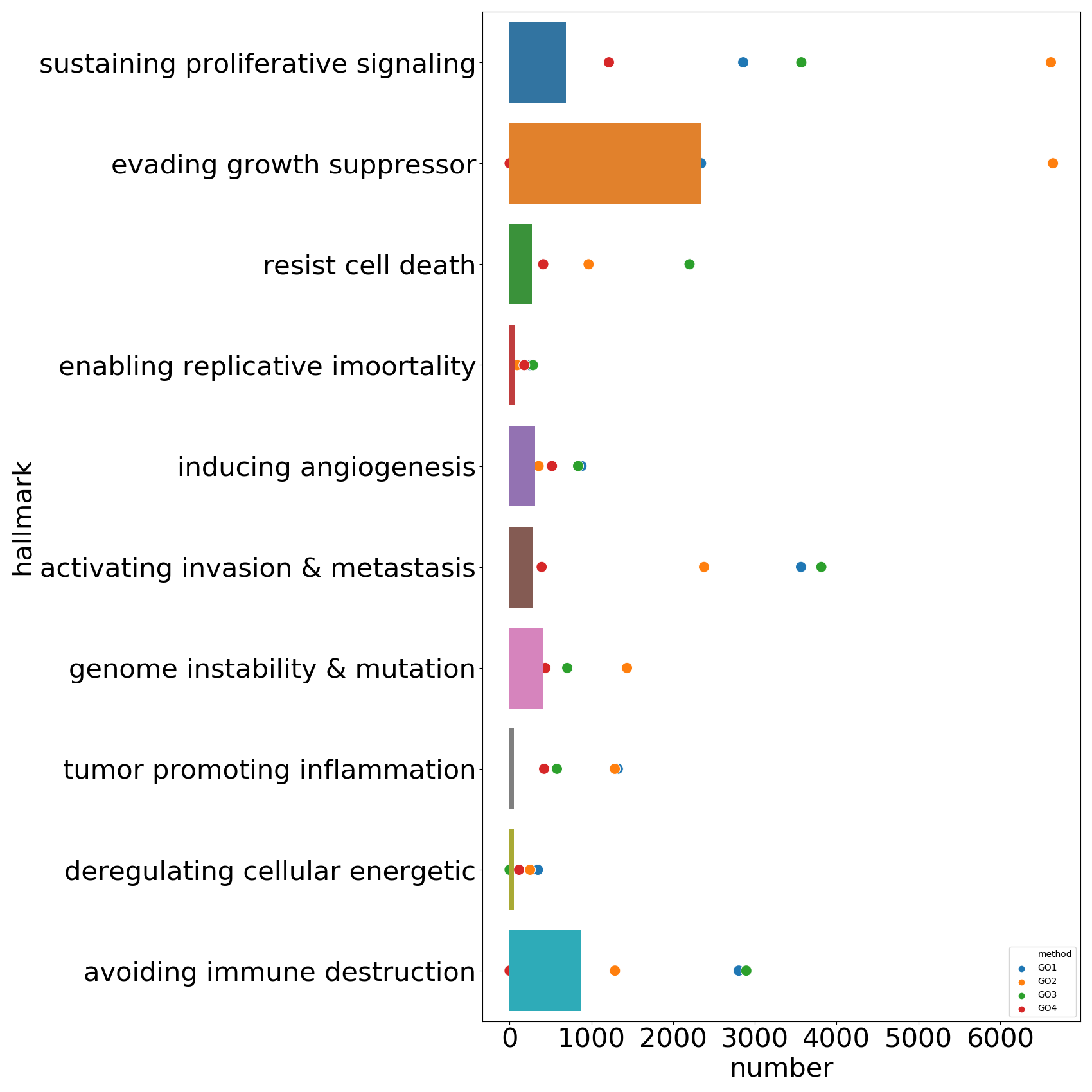

Supplement: Supplementary file 2 — Additional file 2. The genes belong to individual cancer hallmarks with different mapping schemes and their intersections. The dots represent the number of genes attributed to individual cancer hallmarks and the bars represent intersections of all mapping schemes. [file 12859_2021_4105_MOESM2_ESM.png]

# Cluster Dendrogram

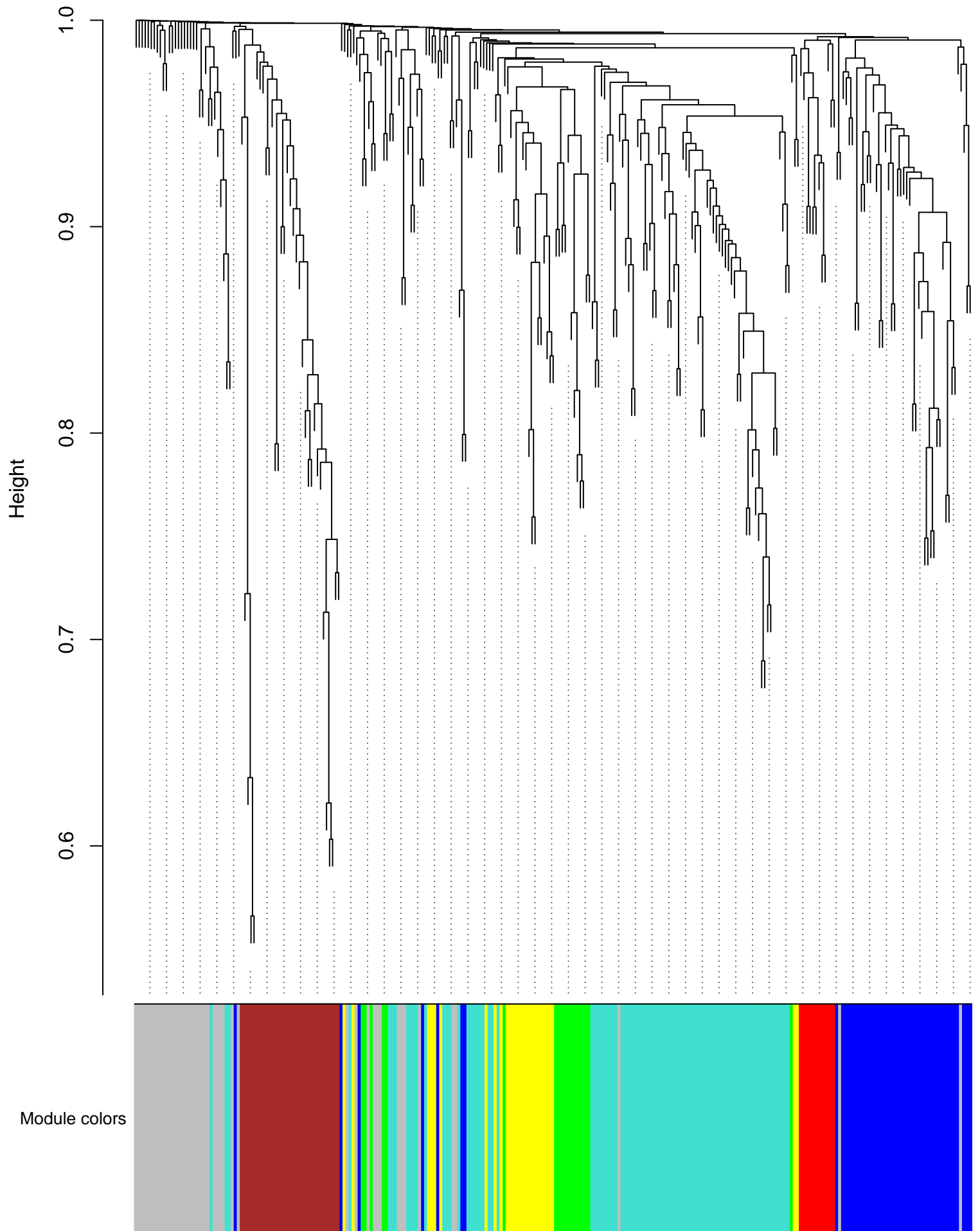

Cluster Dendrogram

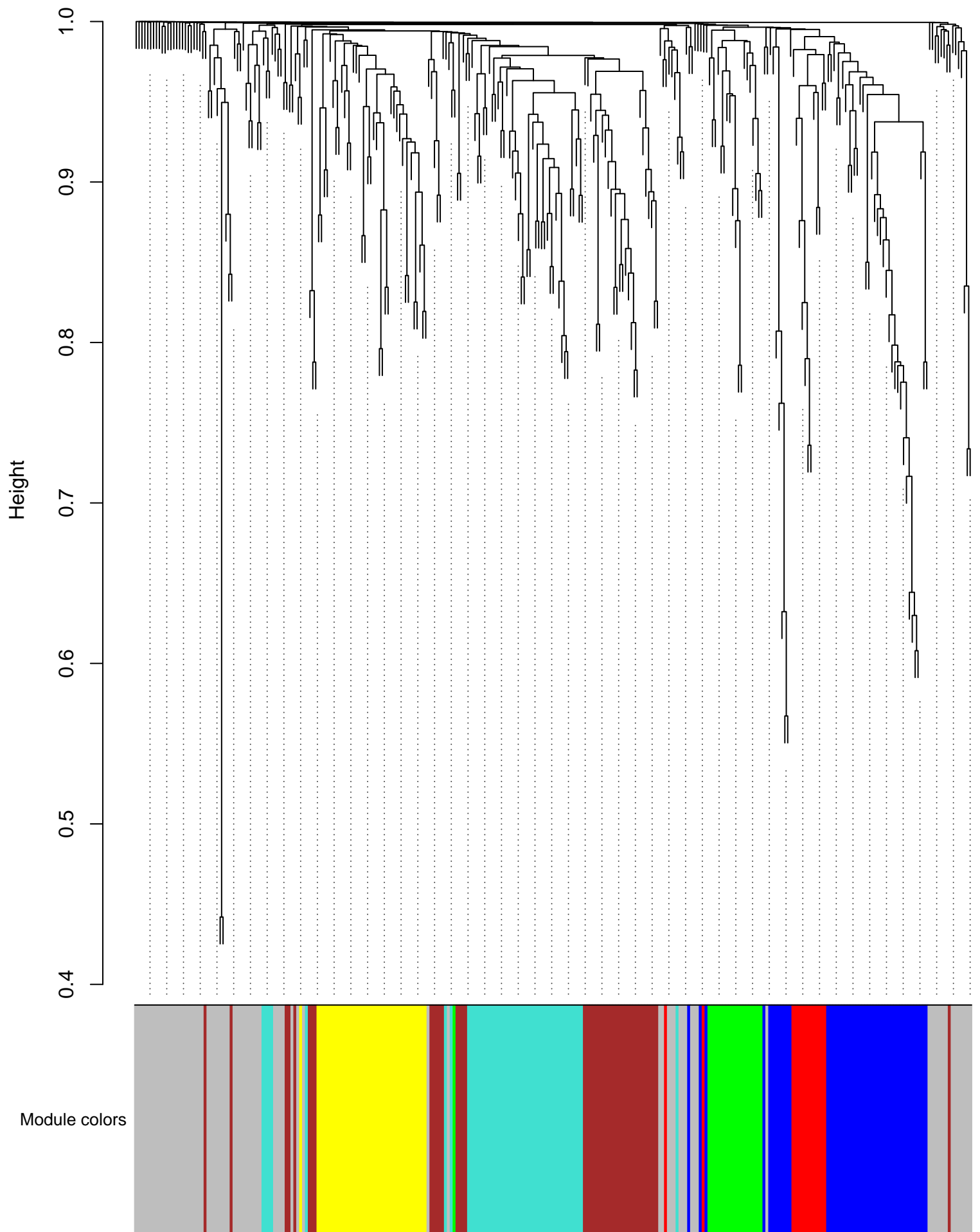

Cluster Dendrogram

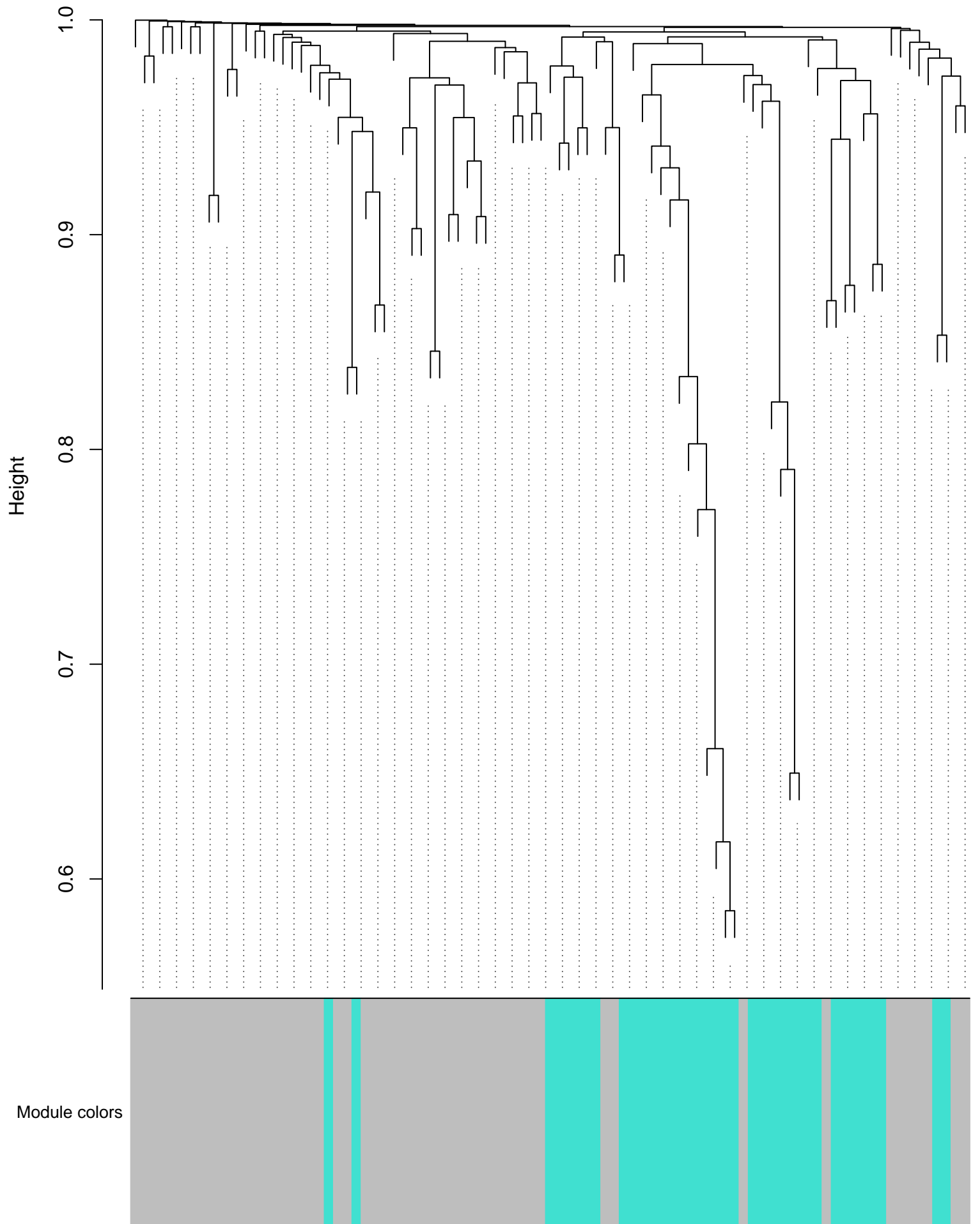

Supplement: Supplementary file 4 — Additional file 4a–c. The cluster dendrograms of prognostic hallmark gene sets from GO2, GO3 and GO4. Prognostic hallmark genes are clustered into different modules based on their co-expression similarity. [file 12859_2021_4105_MOESM4_ESM.pdf]

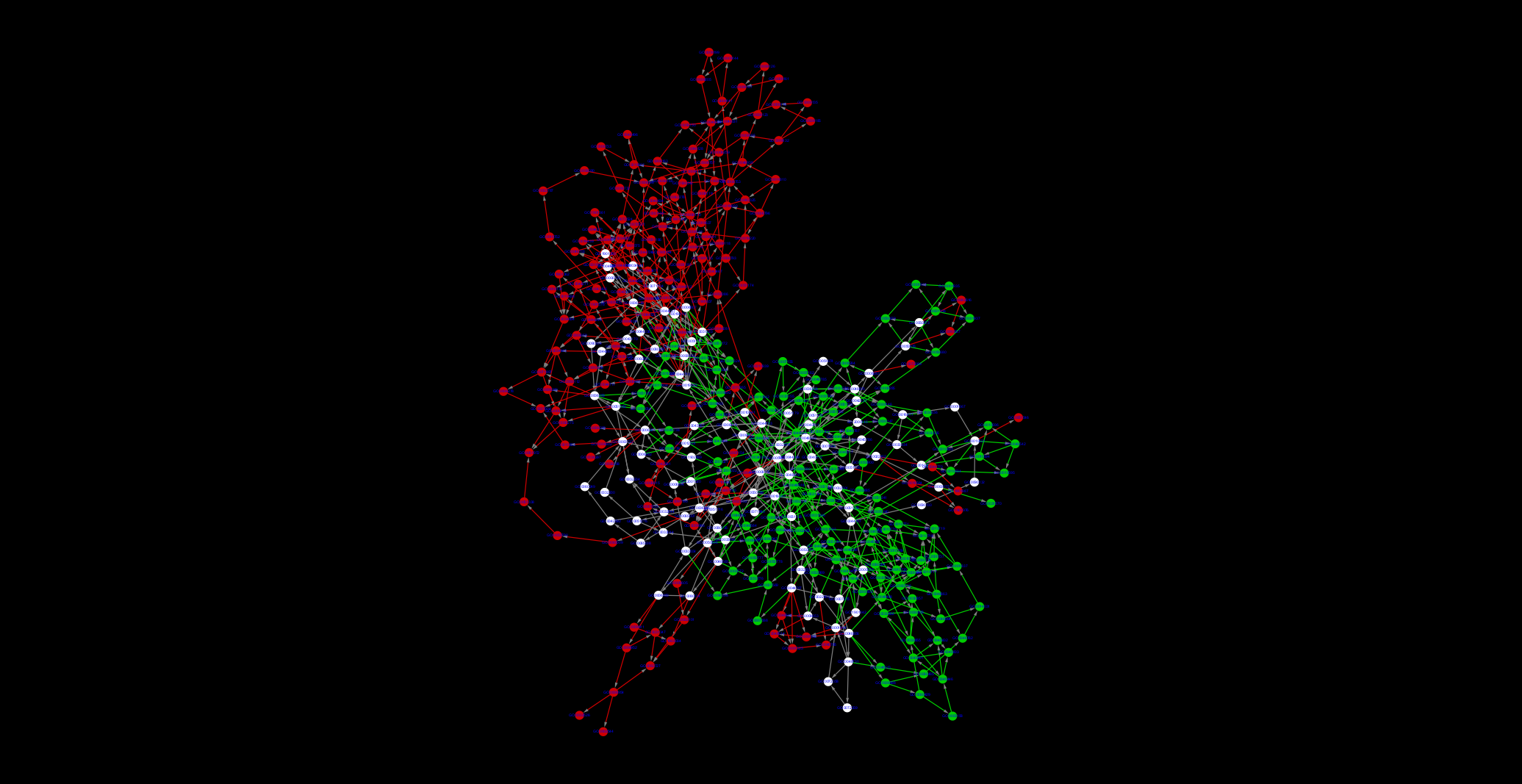

Supplement: Supplementary file 6 — Additional file 6. Network comparison between GO3 and GO4. The two networks were constructed by utilizing GO archived term-to-term relationship data. Network comparison was accomplished by using Dynet. Green nodes are GO terms only selected by GO3 while red nodes are GO terms only selected by GO4. [file 12859_2021_4105_MOESM6_ESM.png]

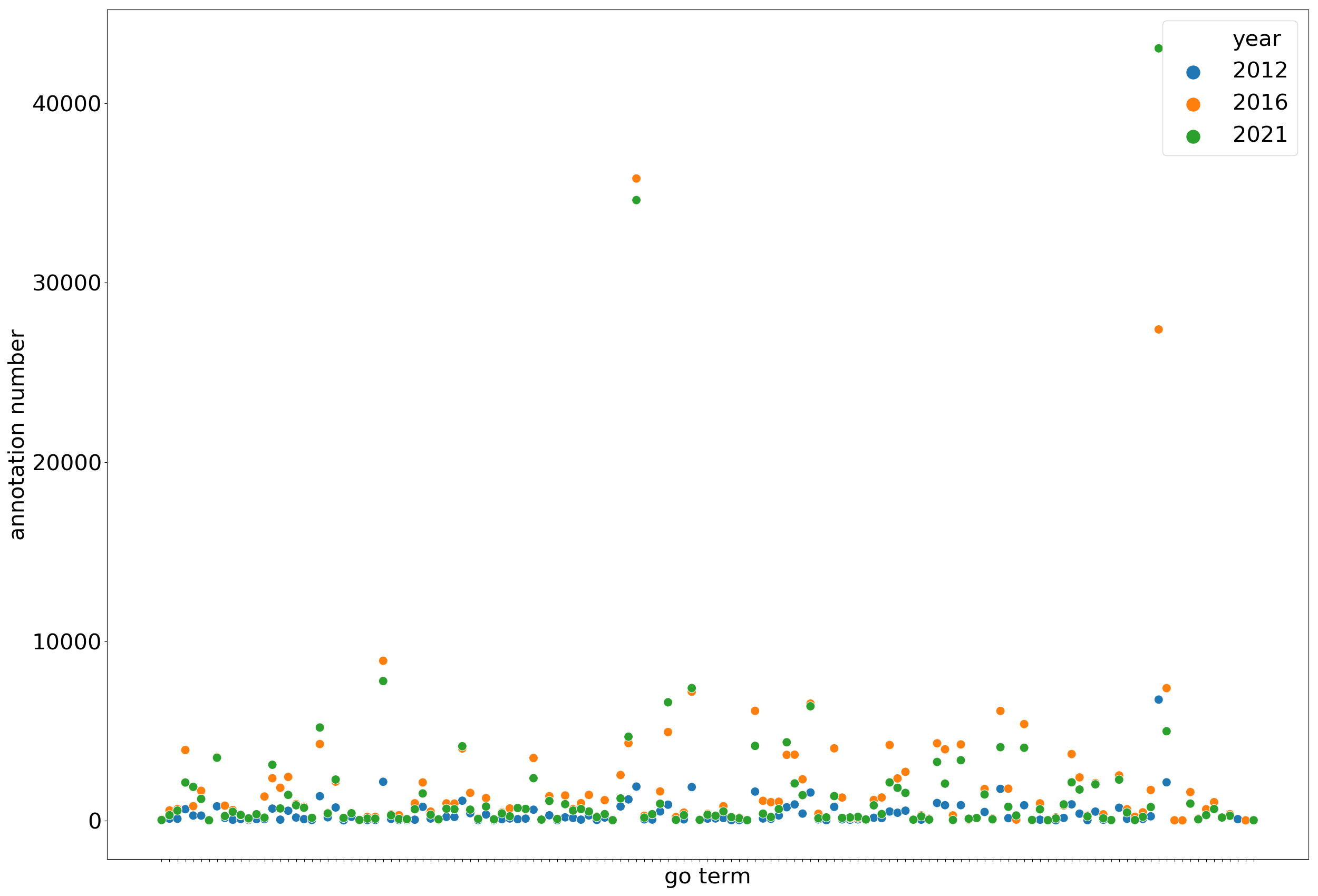

Supplement: Supplementary file 7 — Additional file 7. The annotation counts of GO terms at different GO version. It presents annotation counts of all selected GO terms at 3 time points (2012,2016,2021). [file 12859_2021_4105_MOESM7_ESM.png]

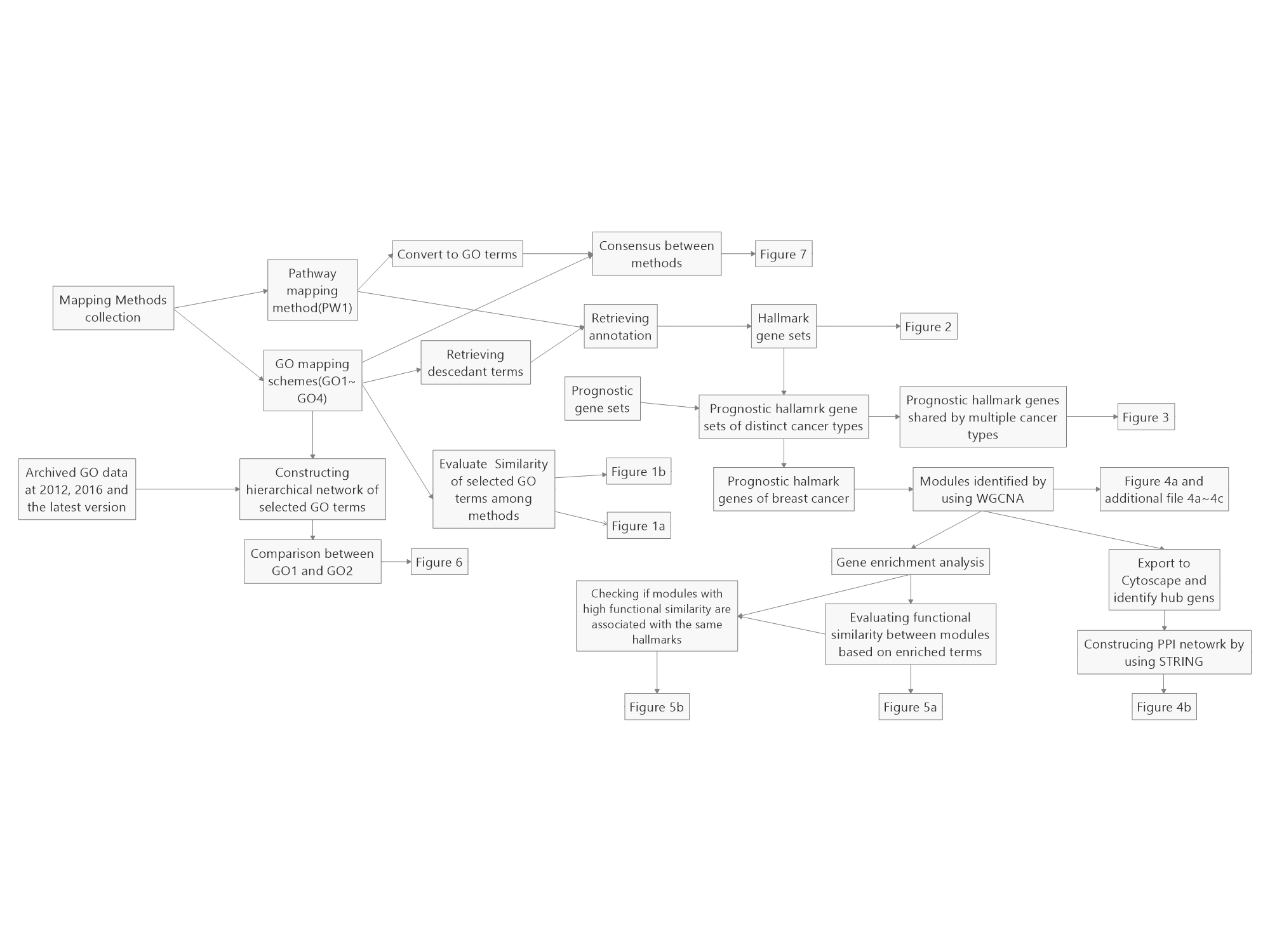

Supplement: Supplementary file 10 — Additional file 10. Study workflow. It can be divided into 2 parts. The first part presents the comparison between different mapping methods and the investigation of the impact of using different mapping schemes on downstream analysis. The second part shows the process of investigating the impact of GO evolutions on the differences between mapping schemes. [file 12859_2021_4105_MOESM10_ESM.png]
